# Oral delivery of GLP-1 peptide using recombinant *Lactobacillus gasseri* for the treatment of type 2 diabetes mellitus

**DOI:** 10.1128/spectrum.02828-24

**Published:** 2025-06-18

**Authors:** Zhiqiang Ke, Qianqian Ma, Xiaonan Ye, Yan Jin, Yanlin Wang, Xinyuan Zhao, Zhengding Su

**Affiliations:** 1 Protein Engineering and Biopharmaceuticals science, Hubei University of Technology 47896, Wuhan, China; 2 Institute of Materia Medica, School of Pharmaceutical Sciences, Xinjiang University 47907, Urumqi, China; 3 National Demonstration Center for Experimental General Medicine Education, School of Basic Medical Sciences, Hubei University of Science and Technology 662810, Xianning, China; 4 Wuhan Biodevelop Inc., Wuhan, China; University of Mississippi, University, Mississippi, USA

**Keywords:** glucagon-like peptide-1, advanced microbiome delivery, protein transduction domain, serum albumin binding peptide, oral delivery, GLP-1 analog, type 2 diabetes mellitus, *Lactobacillus gasseri*, gut microbiota

## Abstract

Glucagon-like peptide-1 (GLP-1) is an effective therapeutic peptide for the treatment of type 2 diabetes mellitus (T2DM). Here, we constructed an effective probiotic-based oral GLP-1 delivery system by engineering the probiotic strain of *Lactobacillus gasseri* (*Lgs*^GPA^) to secrete GLP-1 fusion peptide, which combines GLP-1 with protein transduction domain (PTD) and a serum albumin binding peptide (ABP), GLP-1-PTD-ABP (GPA). The supernatants of *Lgs*^GPA^ cultures significantly upregulated the expression of PDX-1 and stimulated insulin release in Min6 cells. Daily oral administration of *Lgs*^GPA^ in *db/db* mice significantly alleviated insulin resistance, hyperglycemia, and dyslipidemia. Simultaneously, the abundance of *unclassified_f_Erysipelotrichaceae* and *Intestinimonas* was significantly reduced in *db/db* mice, while the average abundance of *Akkermansia* increased in the SD rats. These findings demonstrate that the probiotic-based delivery system represents a versatile and effective strategy for the oral administration of therapeutic peptides. Collectively, our results highlight the potential of this probiotic-based approach as a promising therapeutic and preventive intervention for T2DM.

**IMPORTANCE:**

It is important to develop the oral delivery strategy for therapeutic peptides. Due to issues with patient adherence and the low oral bioavailability of current administration methods, researchers have been exploring oral delivery strategies for GLP-1 analogs for many years, including the use of advanced microbiome therapeutics (AMTs). AMTs offer the potential to use engineered microbes for innovative therapeutic applications, such as the oral delivery of GLP-1 analogs. Our approaches offer a general oral delivery strategy for therapeutic peptides. The probiotic-based approach represents a promising method for treating and preventing T2DM.

## INTRODUCTION

Diabetes mellitus was identified in 20% of patients who experienced their first myocardial infarction (MI) at or before the age of 50, and its presence was associated with significantly worse long-term outcomes, including increased all-cause and cardiovascular mortality (1). Patients with type 2 diabetes mellitus (T2DM) and a history of MI are at high risk for major adverse cardiovascular events (MACE) and cardiovascular death/hospitalization for heart failure. Even among asymptomatic adults, poorer glycemic control is linked to a higher prevalence and severity of coronary atherosclerosis, high-risk plaque, and stenosis (2). Furthermore, even among asymptomatic adults, poorer glycemic control is linked to a higher prevalence and severity of coronary atherosclerosis, high-risk plaque, and stenosis (3). These findings underscore the critical need for more aggressive therapeutic interventions to mitigate the risk of future adverse cardiovascular events in this high-risk population. Glucagon-like peptide-1 (GLP-1) receptor agonists have demonstrated efficacy in the management of T2DM, with robust evidence supporting their cardiovascular benefits (4). However, a significant limitation of most GLP-1 receptor agonists is their peptide-based structure, which necessitates administration via injection. Semaglutide (Rybelsus) represents a notable advancement as the first and only orally available GLP-1 receptor agonist, despite its challenges, including low bioavailability and a high incidence of gastrointestinal side effects (5, 6). To address these issues, new strategies for the oral delivery of GLP-1 receptor agonists are needed to enhance patient adherence and therapeutic outcomes.

Liraglutide is an acylated GLP-1 analog with 97% amino acid sequence homology to native GLP-1, coupled with a substantially prolonged duration of action (7). In prior research, Cheng et al. developed an innovative approach to engineer a precursor peptide for liraglutide, specifically a mutant form of GLP-1 (GLP-1 (7–37)^R34^) (8). Human serum albumin (HSA), an endogenous molecular transporter, is widely recognized for its favorable properties, including excellent biodegradability, non-toxicity, and non-immunogenicity. With a half-life of approximately 19 days, HSA is frequently utilized as a drug carrier to enhance the pharmacokinetic profile and extend the half-life of therapeutic peptides (9). For instance, albiglutide, a GLP-1 analog, is fused with HSA to achieve prolonged therapeutic effects (10). In an earlier study, Li et al. identified a panel of serum albumin-binding peptides (ABPs) with high affinity for HSA through biopanning of a phage display library (11). In addition, protein transduction domains (PTDs) possess intrinsic transmembrane transport capabilities, enabling PTD-modified nanoparticles to facilitate cellular uptake via endocytosis or osmosis (12). Notably, it has been demonstrated that the presence of histidine at position 7 of GLP-1, particularly as a free N-terminal amino acid, plays a critical role in mediating the insulinotropic activity of GLP-1 (13). Therefore, in this study, the ABP and PTD were strategically incorporated into the C-terminal region of the fusion peptide to optimize its functional properties.

The gut microbiota plays a crucial role in regulating host physiological and pathophysiological processes (14). Vowst, a novel oral probiotic microbiota transplantation drug, has been approved by the United States Food and Drug Administration (FDA) as a prophylactic therapy to prevent recurrent *Clostridioides difficile* infections (CDIs) (15, 16). Probiotic microbial transplantation can improve insulin sensitivity in patients with severe obesity and metabolic syndrome, and supplementation with *Akkermansia muciniphila* has shown improvements in metabolic parameters, including insulin sensitivity, reduced insulinemia, and lower plasma total cholesterol (17). The probiotic *Lactobacillus gasseri (L. gs*) SBT2055 has been shown to enhance insulin secretion in Goto-Kakizaki rats by reducing inflammation (18). Due to issues with patient adherence and the low oral bioavailability of current administration methods, researchers have been exploring oral delivery strategies for GLP-1 analogs for many years (19), including the use of advanced microbiome therapeutics (AMTs) (20). AMTs offer the potential to use engineered microbes for innovative therapeutic applications, such as the oral delivery of GLP-1 analogs. Probiotics have potential as an oral delivery of peptides for chronic inflammatory disorders, metabolic diseases (21), obesity (22), and diabetes (23). The secretory signal peptide represents a universal protein sorting signal that is cleaved by signal peptidases following the completion of its targeting function (24). Protein secretion is widely regarded as a preferred mechanism for protein expression in the development of *lactic acid* bacteria as cellular factories for the production of biologically active compounds (25, 26). Among the various signal peptides, usp45, derived from the major secreted protein of *Lactococcus lactis*, has been extensively utilized in genetic engineering strategies to facilitate the secretion of target proteins of interest (27). The secretory signal peptide is a ubiquitous protein sorting signal that is removed by signal peptidases once its targeting function has been carried out (24). Therefore, in this study, the usp45 was strategically incorporated into the N-terminal region of the fusion peptide to facilitate efficient secretion.

In this study, we designed and developed a novel fusion peptide, GLP-1-PTD-ABP (GPA), engineered to traverse the intestinal barrier and extend its plasma half-life. The bioactivity of this peptide was validated through *in vivo* experiments. Subsequently, we engineered a probiotic strain of *L. gs* to secrete GPA peptide (*Lgs*^GPA^) and evaluated its bioactivity using both *in vitro* and *in vivo* models. In addition, we investigated the impact of *Lgs*^GPA^ on the composition and function of the gut microbiota. Our findings demonstrate a promising and innovative strategy for the oral delivery of therapeutic peptides, highlighting its potential for clinical applications.

## RESULTS

### Preparation and characterization of recombinant GPA peptide

**Fig 1:**
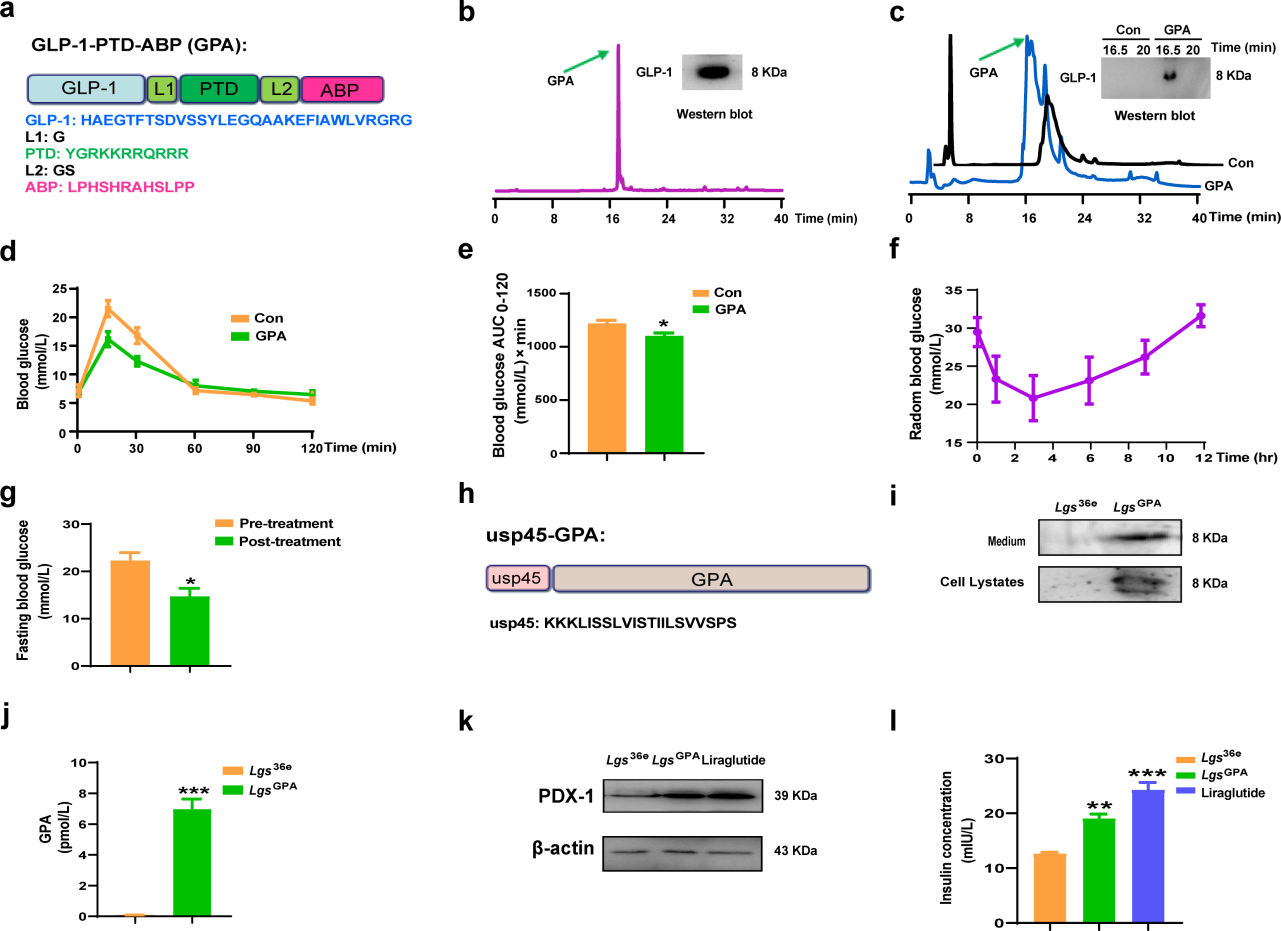
Preparation of GPA peptide and detection of its bioactivity *in vivo* and *in vitro*. (**a**) The schematic of the GPA gene cassette subcloned into the pMFH vector. The amino acid sequences of GLP-1, PTD, and ABP and two linkers, L1 and L2. (**b**) HPLC profile of purified GPA peptide peak with a retention time between 16.5 and 17.0 min was collected and confirmed by Western blot analysis. (**c**) The detection of GPA peptide in the blood by HPLC and Western blot analysis, after the rats were injected with 0.02 mg/kg of body weight GPA for 24 h. (**d and e**) Blood glucose levels (**d**) and the area under the curve (AUC) (**e**) in the glucose tolerance test (GTT) of C57BL/6J mice after intraperitoneal injection of 0.2 mg/kg of body weight GPA peptide for 24 h. (**f**) Random blood glucose of *db/db* mice (*n* = 6) after intraperitoneal injection of 0.2 mg/kg of body weight GPA peptide. (**g**) Fasting blood glucose of *db/db* mice (*n* = 6) after intraperitoneal injection of 0.2 mg/kg of body weight GPA peptide for 24 h. (**h**) The schematic of the gene cassette subcloned into the pMG36e vector. (**i and j**) The GPA peptide released to the supernatant was identified by Western blot analysis (**i**) and enzyme-linked immunosorbent assay (ELISA) (**j**), respectively. (**k and l**) The β-cell markers PDX-1 (**k**) and insulin release (**l**) by Min6 cells, respectively, in the presence of medium of recombinant *L. gs*. Data were presented as means ± SEM. *P < 0.05, **P < 0.01, ***P < 0.001, determined by unpaired two-tailed Student’s t test for two-group comparisons, one-way ANOVA followed by Tukey’s multiple comparison test for multiple group comparisons.

In this study, the plasmid pUC57-usp45-GPA was commercially synthesized. The target gene fragment encoding GPA was subsequently cloned into the pMFH vector, as illustrated in Fig. 1a and Fig. S1a and b. The resulting pMFH-GPA plasmid was then transformed into *E. coli* BL21 for expression. Following fermentation, the expression of pMFH-GPA fusion protein was induced by Isopropyl β-D-thiogalactoside (IPTG) and confirmed through tricine-SDS–PAGE and Western blot analysis (Fig. S1c and d). The pMFH-GPA fusion protein, with a molecular weight of 22 kDa (Table S3), has a theoretical isoelectric point (pI) of 9.83, as determined using the ExPASy ProtParam tool (https://web.expasy.org/protparam/). The fusion protein was subsequently cleaved using cyanogen bromide (CNBr) and purified. The crude GPA peptide was further purified by high-performance liquid chromatography (HPLC) and validated by Western blot analysis (Fig. 1b). The purified GPA peptide has a molecular weight of 8.1 kDa and a theoretical pI of 10.93, as detailed in Table S3.

### Analysis of the GPA peptide and its bioactivity *in vivo*

To evaluate the bioactivity of GPA peptide in rats, the presence of GPA in the blood was analyzed using HPLC and Western blot analysis 24 h after intraperitoneal administration of 0.02 mg/kg of body weight GPA (Fig. 1c). To further assess the bioactivity of GPA peptide in mice, a glucose tolerance test (GTT) was conducted in C57BL/6J mice. Compared to the control group, GPA administration significantly improved blood glucose tolerance and reduced AUC value (*P* < 0.05, Fig. 1d and e).

To further evaluate the bioactivity of GPA peptide in *db/db* mice, random blood glucose levels were measured. As illustrated in Fig. 1f, the random blood glucose levels in *db/db* mice were decreased gradually within 3 h post-injection, followed by a gradual increase, returning to the baseline levels by 12 h. In addition, fasting blood glucose levels were monitored in *db/db* mice before and after treatment with 0.2 mg/kg body weight GPA peptide. As shown in Fig. 1g, the peptide significantly reduced fasting blood glucose levels 24 h after administration. These findings demonstrate the glucose-lowering effects of the GPA peptide in both normoglycemic and diabetic murine models.

### Detection of GPA peptide secreted by *Lgs*^GPA^ and its bioactivity *in vitro*

The target gene fragment encoding usp45-GPA was cloned into the pMG36e vector (Fig. 1h; Fig. S2 and S3), enabling the successful construction of the recombinant probiotic strain, *Lgs*^GPA^. As a control, a recombinant probiotic strain harboring the empty pMG36e vector, *Lgs*^36e^, was also generated (Fig. S4).

The expression of the GPA peptide in the supernatant of the recombinant *L. gs* strains was confirmed by Western blot analysis (Fig. 1i) and quantified using an ELISA assay (Fig. 1j). To assess the bioactivity of the secreted GPA peptide, the expression of β-cell markers PDX-1 and insulin release were evaluated in Min6 cells treated with the medium of the recombinant *L. gs* strains. Liraglutide was used as a positive control. Compared with the *Lgs*^36e^ group, the *Lgs*^GPA^ group exhibited a significant increase in the expression of PDX-1 (Fig. 1k). As shown in Fig. 1l, although the insulin secretion induced by the medium of *Lgs*^GPA^ was slightly less pronounced than that induced by 100 nmol/L liraglutide, it still significantly stimulated insulin secretion compared to the medium of *Lgs*^36e^ (*P* < 0.01). These results demonstrate the functional activity of the GPA peptide secreted by *Lgs*^GPA^ in promoting β-cell marker expression and insulin release *in vitro*.

### Bioactivity of *Lgs*^GPA^ in *db/db* mice

**Fig 2:**
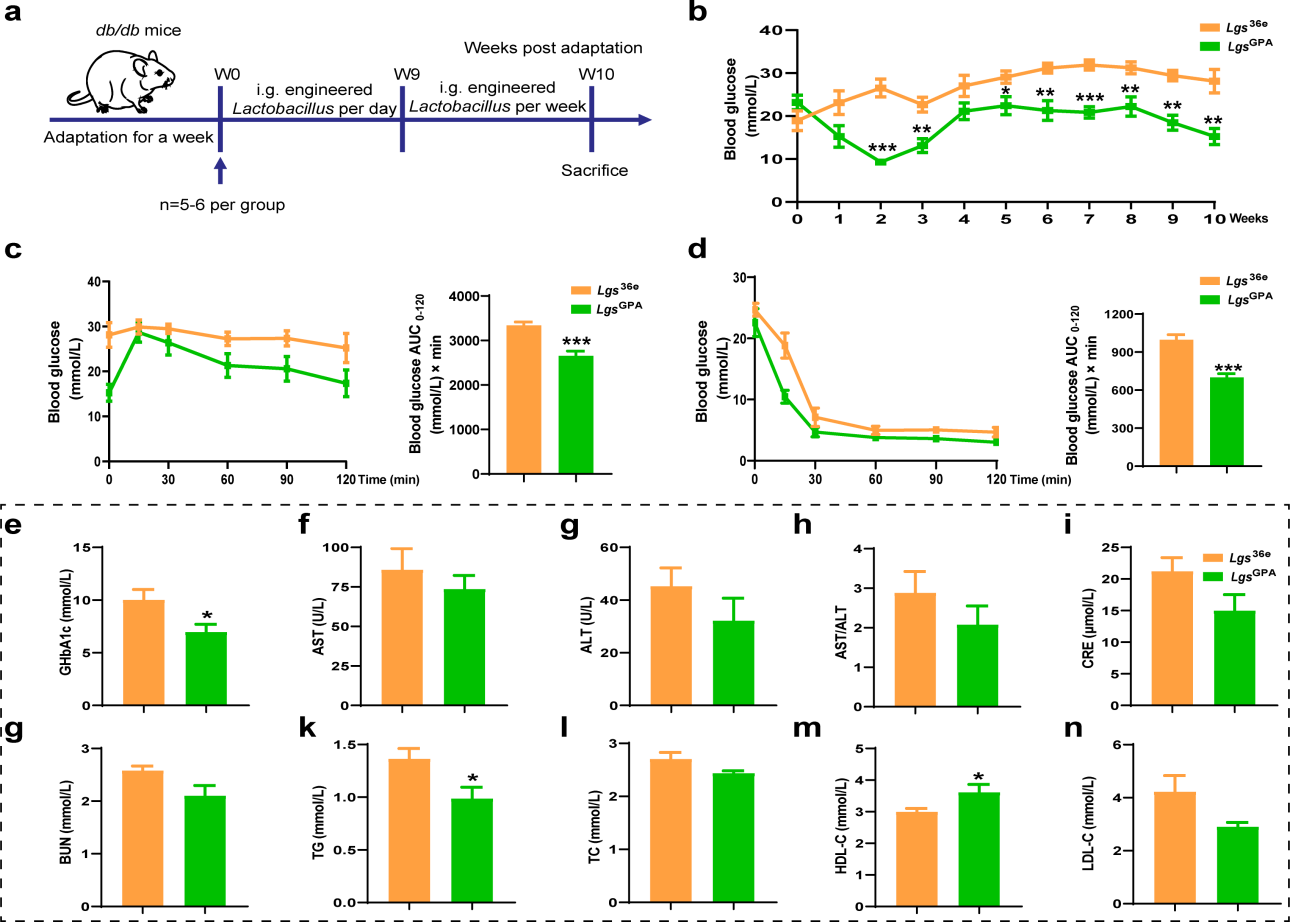
Improved the effects of *Lgs*^GPA^ on diabetic symptoms in *db/db* mice. (**a**) The schematic of the animal experiment. (**b**) Fasting blood glucose levels. (**c**) Blood glucose levels and the area under the curve (AUC) in the glucose tolerance test (GTT) of the *db/db* mice. (**d**) Blood glucose levels and the AUC in the insulin tolerance test (ITT) of the *db/db* mice. (**e**) Serum levels of glycosylated hemoglobin A1c (GHbA1c). (**f**) Serum levels of aspartate aminotransferase (AST). (**g**) Serum levels of alanine aminotransferase (ALT). (**h**) The ratio of AST/ALT. (**i**) Serum levels of creatinine (CRE). (**j**) Serum levels of urea (BUN). (**k**) Serum levels of triglyceride (TG). (**l**) Plasma levels of total cholesterol (TC). (**m**) Serum levels of high-density lipoprotein cholesterol (HDL-C). (**n**) Serum levels of low-density lipoprotein cholesterol (LDL-C). *Lgs*^36e^ group, the *db/db* mice were administered *Lgs*^36e^ (*n* = 6); *Lgs*^GPA^ group, the *db/db* mice were administered *Lgs*^GPA^ (*n* = 5). Data were presented as means ± SEM. *P < 0.05, **P < 0.01, ***P < 0.001 *vs Lgs*^36e^ group, determined by unpaired two-tailed Student’s t test for two-group comparisons.

To evaluate the therapeutic potential of *Lgs*^GPA^, its bioactivity was investigated in *db/db* mice. Compared to the *Lgs*^36e^ group, the fasting blood glucose levels in the *Lgs*^GPA^ group exhibited a decreasing trend starting from the 2nd week. Although this reduction was not statistically significant during the 4th and 5th weeks, by the 6th week, the fasting blood glucose levels in the *Lgs*^GPA^ group were significantly lower than those in the *Lgs*^36e^ group (Fig. 2b). Furthermore, *Lgs*^GPA^ significantly improved glucose tolerance, as evidenced by a notable reduction in the AUC value (*P* < 0.001), compared to the *Lgs*^36e^ group (Fig. 2c). A similar trend was observed in the insulin tolerance test (ITT), with the AUC values mirroring those of the GGT (Fig. 2d). In addition, *Lgs*^GPA^ significantly reduced serum levels of glycosylated hemoglobin A1c (GHbA1c) compared to the *Lgs*^36e^ group (Fig. 2e).

To assess the broader metabolic effects of *Lgs*^GPA^, various blood biochemical parameters were measured in *db/db* mice. As shown in Fig. 2, *Lgs*^GPA^ had no significant impact on liver function, as indicated by serum levels of aspartate aminotransferase (AST) (Fig. 2f), alanine aminotransferase (ALT) (Fig. 2g), and the ratio of AST/ALT (Fig. 2h). Similarly, kidney function, measured by serum creatinine (CRE) (Fig. 2i) and blood urea nitrogen (BUN) levels (Fig. 2j), remained unaffected. *Lgs*^GPA^ also did not alter serum levels of total cholesterol (TC) (Fig. 2l) or low-density lipoprotein cholesterol (LDL-C) (Fig. 2n). However, compared to the *Lgs*^36e^ group, *Lgs*^GPA^ significantly increased serum levels of high-density lipoprotein cholesterol (HDL-C) (Fig. 2m) (*P* < 0.05) and significantly decreased serum levels of triglyceride (TG) (Fig. 2k) (*P* < 0.05). These findings highlight the potential of *Lgs*^GPA^ to improve glycemic control and lipid metabolism in a diabetic mouse model without adversely affecting liver or kidney function.

### Effect of *Lgs*^GPA^ on the gut microbial community of the type 2 diabetic mouse model

**Fig 3:**
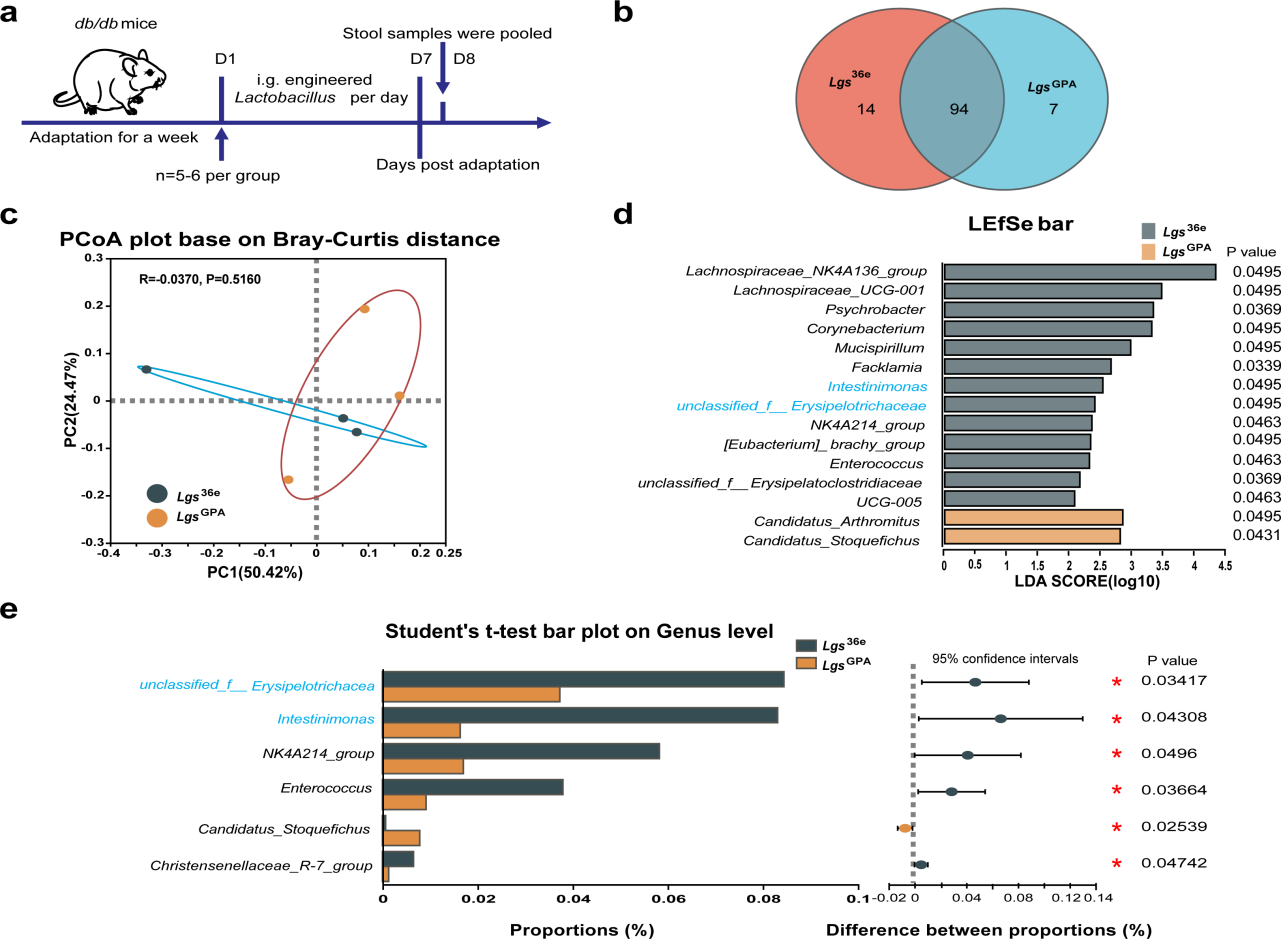
*Lgs*^GPA^ remodels the gut microbiome in *db/db* mice. (**a**) The schematic of the study stool sample collection of each group at the indicated time points. (**b**) The total number of core operational taxonomic units (OTUs) shared and that are unique in the Venn diagram. (**c**) β diversity analysis of PCoA analysis on the OTU level. (**d**) Linear discriminant analysis (LDA) effect size (LEfSe) analysis to identify bacterial genera whose abundance differed significantly between the two groups of mice. Only taxa with LDA scores of more than 2 were presented (*n* = 3). *P* value was determined by the Mann-Whitney U test. (**e**) Student’s t-test bar plot on genus level.

To explore the therapeutic potential of *Lgs*^GPA^, its impact on the gut microbiome of *db/db* mice was analyzed. A total of 376,173 high-quality reads and 1,717 operational taxonomic units (OTUs) were obtained. The α diversity analysis, including the ace index, sobs index, Chao1 index, Shannon index, Pielou evenness, and Simpson index at the OTU level, revealed no significant differences between the *Lgs*^GPA^ and *Lgs*^36e^ groups (Fig. S6a). The Venn diagram revealed 94 common OTUs between the two groups, with 14 unique OTUs in the *Lgs*^36e^ group and 7 unique OTUs in the *Lgs*^GPA^ group, respectively (Fig. 3b). The β diversity analysis of principal coordinates (PCoA) demonstrated partial separation between the two groups (Fig. 3c).

**Fig 4:**
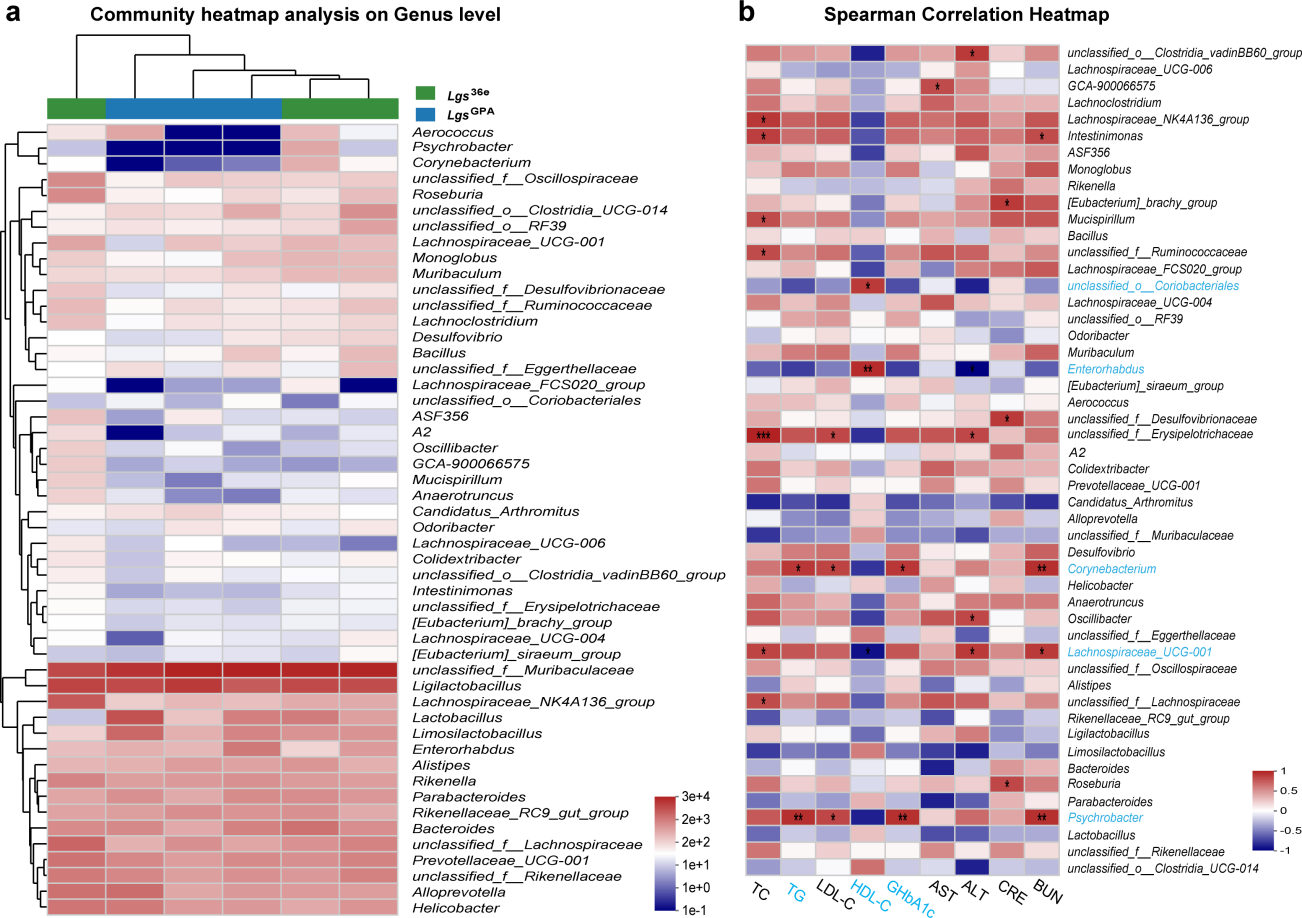
The analysis at the genus level on the stool microbiome of *db/db* mice. (**a**) The community heatmap analysis of the top 50 taxonomic composition analysis and the abundance at the genus level. (**b**) The correlation heatmap analysis of gut microbiota with clinical factors.

Taxonomic analysis revealed differences in microbial composition between the groups. At the phylum level, *Firmicutes* and *Bacteroidota* were dominant taxa. In the *Lgs*^GPA^ group, the average relative abundance of *Firmicutes* decreased, while that of *Bacteroidota* increased, resulting in a reduction of the *Firmicutes/Bacteroidota* (F/B) ratio, although these changes were not statistically significant (Fig. S5a and S6b). At the order level, *Bacteroidales*, *Lactobacillales,* and *Lachnospirales* were predominant. The relative abundance of *Clostridiales* (*P* = 0.09) increased, while *Desulfovibrionales* (*P* = 0.08) and *Erysipelotrichales* (*P* = 0.08) decreased in the *Lgs*^GPA^ group compared to the *Lgs*^36e^ group (Fig. S5b and S6c). At the family level, *Muribaculaceae*, *Lactobacillaceae*, and *Rikenellaceae* were the most abundant. Notably, the relative abundance of *Erysipelotrichaceae*, *Enterococcaceae*, and the combined abundance of these two potentially pernicious bacteria was significantly reduced (*P* < 0.05) in the *Lgs*^GPA^ group (Fig. S5c and S6d) compared to the *Lgs*^36e^ group. At the genus level, *unclassified_f__Muribaculaceae*, *Ligilactobacillus,* and *Lactobacillus* were predominant (Fig. S5d). The relative abundances of *unclassified_f__Erysipelotrichaceae* and *Intestinimonas* were significantly lower (*P* < 0.05) in the *Lgs*^GPA^ group (Fig. 3e and 4a) compared to the *Lgs*^36e^ group.

Correlation analysis identified three bacterial taxa associated with two clinical parameters (highlighted in red boxes). At the genus level, *unclassified_o__Coriobacteriales*, *Enterorhabdus*, *Corynebacterium*, *Lachnospiraceae_UCG-001*, and *Psychrobacter* (marked in blue) were identified as potential targets of *Lgs*^GPA^ in ameliorating hyperlipidemia or hyperglycemia in *db/db* mice (Fig. 4b).

Linear discriminant analysis effect size (LEfSe) analysis was utilized to estimate microbiome differences at the genus level between the two groups (Fig. 3d). Only taxa with linear discriminant analysis (LDA) scores above 2 were presented, revealing 15 differentially abundant bacteria between the two groups. In addition, Student’s t-test identified six bacteria that differed between the groups, with two (marked in blue) ranking among the top 50 (Fig. 3e), suggesting that the *Lgs*^GPA^ may improve intestinal conditions in *db/db* mice.

Functional predictions based on the 16S amplicon sequencing data, combined with the Clusters of Orthologous Genes (COGs) and KEGG databases, indicated that bacterial functions were primarily related to metabolic pathways (Fig. S7). The COGs database predicted that bacterial functions were predominantly involved in metabolism, with amino acid metabolism being the third most abundant (Fig. S7a). Similarly, 16S rRNA sequencing data combined with KEGG functional predictions indicated that bacterial colony function is primarily related to metabolic pathways, especially carbohydrate metabolism (Fig. S7b and c). Further analysis using KEGG data at a deeper level (3rd Level) confirmed the association of bacterial functions with metabolic pathways (Fig. S7d). These findings suggest that *Lgs*^GPA^ modulates the gut microbiota in *db/db* mice, potentially contributing to its therapeutic effects on metabolic dysregulation.

### Bioactivity of *Lgs*^GPA^ on SD rats

**Fig 5:**
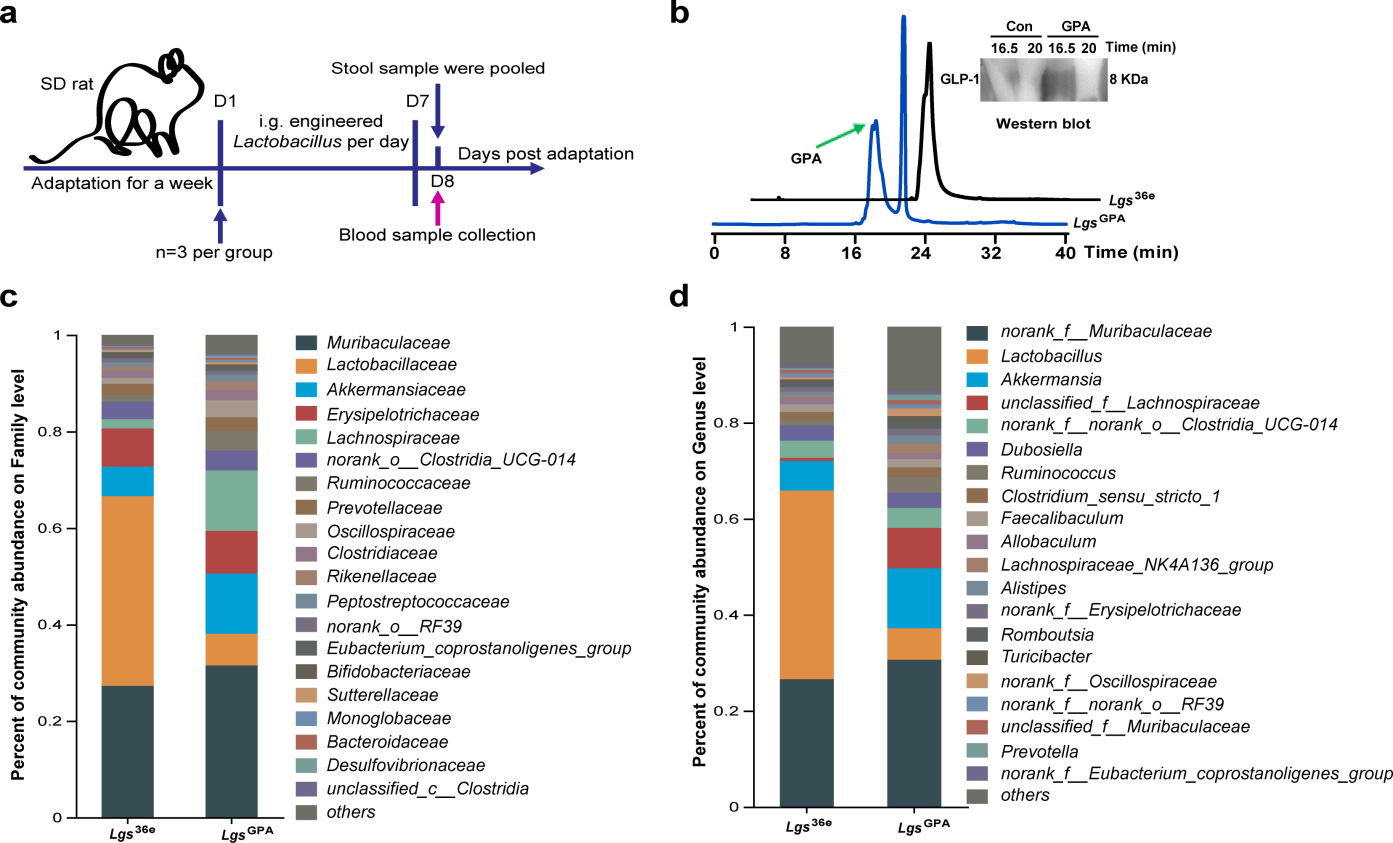
The effects of *Lgs*^GPA^ on the gut microbiome of SD rats. (**a**) The schematic of the study stool and blood sample collection of each group at the indicated time points. (**b**) The content of GPA in the blood of rats was detected by HPLC and Western blot analysis. (**c**) Community bar plot of the most relevant taxa responsible for the difference at the family level. (**d**) Community bar plot of the most relevant taxa responsible for the difference at the genus level.

We confirmed the genetic stability of the recombinant *L. gs* strain across three generations cultured from stool samples using PCR (Fig. S8). As illustrated in Fig. 5a, rats were intragastrically administered the recombinant *L. gs* strains for 1 week. To verify the bioactivity of *Lgs*^GPA^ in the gastrointestinal tract, the presence of the GPA peptide in the blood of rats was analyzed utilizing HPLC and Western blot analysis (Fig. 5b) following intragastric administration of recombinant *Lgs*^GPA^. To further evaluate the potential preventive value of *Lgs*^GPA^, its impact on the stool microbiome of SD rats was investigated. A total of 455,489 high-quality reads and 173 OTUs were obtained. The Venn diagram revealed that 643 common OTUs were shared between the two groups, with 87 unique OTUs in the *Lgs*^36e^ group and 168 unique OTUs in the *Lgs*^GPA^ group (Fig. S9a). The α diversity analysis, including the ace index, sobs index, Chao1 index, Shannon index, Pielou evenness, and Simpson index at the OTU level, showed no significant difference between the two groups (Fig. S9c). However, the β diversity analysis using PCoA indicated partial separation between the two groups (Fig. S9b). These results suggest that *Lgs*^GPA^ may enhance microbial community diversity.

Taxonomic analysis revealed differences in microbial composition between the groups. At the phylum level, *Firmicutes* and *Bacteroidota* were the dominant taxa. In the *Lgs*^GPA^ group, the average relative abundance of *Firmicutes* decreased, while that of *Bacteroidota* increased, resulting in a reduction in the F/B ratio, although these changes were not statistically significant (Fig. S9d). At the family level, *Muribaculaceae*, *Lactobacillaceae,* and *Akkermansiaceae* were the most abundant (Fig. 5c). At the genus level, *norank_f_Muribaculaceae*, *Lactobacillus*, and *Akkermansia* were predominant. Notably, the average relative abundance of *Akkermansia* was higher in the *Lgs*^GPA^ group compared to the *Lgs*^36e^ group, although this increase was not statistically significant (Fig. 5d).

Functional predictions based on 16S amplicon sequencing data, combined with the COGs and KEGG databases, indicated that bacterial functions were primarily associated with metabolic pathways (Fig. S10). The COGs database predicted that bacterial functions were mainly involved in metabolism, with amino acid metabolism representing the third most abundant category (Fig. S10a). Similarly, 16S rRNA sequencing data combined with KEGG functional predictions indicated that bacterial colony function is primarily related to metabolism pathways, particularly carbohydrate metabolism (Fig. S10b and c). Further analysis using KEGG at a deeper level (3rd Level) confirmed the association of bacteria with metabolic pathways (Fig. S10d). These findings suggest that *Lgs*^GPA^ modulates the gut microbiota in SD rats, potentially contributing to its preventive and therapeutic effects through metabolic regulation.

## DISCUSSION

This study demonstrates that the bioactivity of GPA peptide, particularly when secreted by *Lgs*^GPA^, significantly enhances the expression of PDX-1 and stimulates insulin release in Min6 cells. PDX-1, a transcription factor critical for pancreatic development, islet neogenesis, and the maintenance of mature β-cell function, represents a promising therapeutic target for reversing diabetes (28, 29). Although data on food intake were not systematically analyzed, we monitored the average food intake per mouse over a 2-week period and the average water intake per mouse over a 4-week period. While the food intake data exhibited variability, *Lgs*^GPA^ consistently reduced water intake in *db/db* mice compared to the *Lgs*^36e^ control group. Importantly, *Lgs*^GPA^ administration significantly ameliorated insulin resistance, hyperglycemia, and dyslipidemia in *db/db* mice, highlighting its therapeutic potential for managing metabolic abnormalities associated with diabetes.

In the subsequent phase of this study, we evaluated the therapeutic potential of *Lgs*^GPA^ in *db/db* mice, with a particular focus on its impact on gut microbiota composition. At the phylum level, a reduction in the Firmicutes-to-Bacteroidetes (F/B) ratio was observed, a metric commonly associated with obesity (30). At the family level, the *Lgs*^GPA^ group exhibited a significant decrease in the relative abundance of *Erysipelotrichaceae* and *Enterococcaceae* compared to the *Lgs*^36e^ group. *Erysipelotrichaceae* has been linked to metabolic disorders, including diet-induced obesity in both murine models and humans (31), while *Enterococcaceae* is recognized as a risk factor for *Clostridioides* difficile infection (32). At the genus level, the relative abundances of *unclassified_f__Erysipelotrichaceae* and *Intestinimonas* were significantly reduced in the *Lgs*^GPA^ group compared to the *Lgs*^36e^ group. Numerous studies have reported elevated levels of *Erysipelotrichaceae* in obese individuals (33), hypercholesterolemic hamster models (34), and mice fed high-fat or Western diets, further supporting the association between this bacterial family and adverse lipidemic profiles in the host (35). In addition, *Intestinimonas*, which has been shown to increase in abundance in response to a Western diet (36), is associated with obesity, cardiovascular disease, and metabolic syndrome (37). These findings may explain the observed effects of *Lgs*^GPA^, including a significant increase in HDL-C levels and a reduction in TG levels. Collectively, these results demonstrate that microbiota-mediated modulation of glucose and lipid metabolism represents a critical mechanism underlying the therapeutic effects of *Lgs*^GPA^ in *db/db* mice. To further strengthen the manuscript, future work will include enumeration of the population levels of the recombinant *Lactobacillus* in different regions of the gastrointestinal tract.

We further confirmed that *Lgs*^GPA^ successfully colonized the gut of rats, as evidenced by the cultivation of *Lactobacilli* from stool samples and the verification of the genetic construct’s stability in the stool. In addition, we investigated the impact of *Lgs*^GPA^ on the stool microbiome of SD rats. At the genus level, the relative abundance of *Akkermansia* was increased in the *Lgs*^GPA^ group compared to the *Lgs*^36e^ group. Although *Akkermansia muciniphila* has been associated with metabolic diseases (38), it is also recognized for its potential health benefits in humans (39, 40). Two species of *Akkermansia* have been identified: *Akkermansia muciniphila*, isolated from human stool, and *Akkermansia glycaniphila*, isolated from python stool. *A. glycaniphila* has not been detected in the gut microbiota of mammals, including humans and mice (41). Recent studies have reported that *A. muciniphila* ameliorates symptoms of T2DM by stimulating GLP-1 secretion (40, 42, 43). Furthermore, it has been demonstrated that semaglutide (44) and liraglutide (45) increased the abundance of *Akkermansia*. Therefore, we propose a potential synergistic relationship between *A. muciniphila* and GLP-1 in promoting metabolic health. In 16S rRNA sequencing analyses of gut microbiota, the genus *Akkermansia* was found to be increased in *db/db* mice fed milk, as well as in stool microbiota transplantation groups derived from these mice (41), indicating that *Akkermansia* can be detected in *db/db* mice under certain conditions. Although *Akkermansia* was not detected in the *db/db* mice in our study, *Lgs*^GPA^ significantly reduced the abundance of detrimental bacteria, such as *unclassified_f_Erysipelotrichaceae* and *Intestinimonas*, in these mice. This suggests that *Lgs*^GPA^ exerts beneficial effects on the gut microbiota by modulating the composition of harmful bacterial taxa, even in the absence of detectable *Akkermansia*.

In a recent comprehensive review of oral delivery strategies of peptide GLP-1 receptor agonists, a series of studies have demonstrated that advanced microbiome-based delivery systems represent a viable and innovative approach for the effective delivery of peptide-based GLP-1 receptor agonists (46). In the study by Duan et al., engineered *L. gs* ATCC 33323 was designed to secrete an inactive form of GLP-1 (1–37), which primarily functions as a stem cell stimulator, promoting the conversion of intestinal epithelial cells into insulin-secreting cells to secrete insulin in a diabetic rat model (23). By contrast, our peptide construct features an active form of GLP-1, specifically GLP-1 (7–37), fused with an HSA-binding peptide to significantly extend the half-life of GLP-1 (7–37), as demonstrated in our previous work (11). In addition, our peptide incorporates a PTD to assist efficient crossing of the intestinal barrier. As a result, our peptide construct functions as a long-lasting GLP-1 receptor agonist, directly stimulating insulin secretion. In comparison, the construct developed by Duan et al. lacks GLP-1R agonist activity and does not directly induce insulin secretion, instead acting as a modulator of stem cell differentiation. These differences highlight the unique therapeutic potential of our approach compared to the previously published work.

In summary, this study demonstrates the development of an engineered probiotic system for the oral delivery and sustained release of GLP-1 peptide as a therapeutic intervention for T2DM. While further investigation is required to fully elucidate the mechanisms and optimize the therapeutic efficacy, our findings present a promising strategy for the prevention and management of T2DM. This approach highlights the potential of leveraging probiotic-based delivery systems to address the challenges associated with peptide therapeutics in metabolic diseases.

## MATERIALS AND METHODS

### Strains and reagents

For this study, the bacterial strains and plasmids used are given in Table S1, primers from Genscript Co. (Nanjing, China) are given in Table S2. DNA restriction enzymes (*Xba* I, *Pst* I, *Eco*R, and *Bam*H I), *Dpn* I, alkaline phosphatase (Calf intestine, CIAP), and the T4 DNA ligase for ligation were supplied by Takara Co. (Dalian, China). Mini-plasmid kit was from Tiangen Co. (Beijing, China). GLP-1 and pancreatic duodenal homeobox-1 (PDX-1) antibody were from Abclonal Co. (Wuhan, China). Min6 cell line was from Servicebio Co. (Wuhan, China). RPMI Medium 1640 basic and Fetal Bovine Serum (FBS) were from Gibco (New York, USA). Liraglutide was from Aikang Biopharmaceutical (Jiangsu, China). Other chemicals and reagents used were analytical grade.

### Construction of plasmids and recombinant strains

The plasmids pMFH-GPA and pMG36e-usp45-GPA (pMG36e-GPA) were constructed by our lab. All the constructed plasmids were verified with DNA sequencing by Tianyihuiyuan Co. (Wuhan, China). Detailed experimental protocols are described in the supplementary information. The pMFH-GPA fusion gene was transformed into *E. coli* DH5α, *E. coli* BL21 using the heat shock method. The pMG36e-usp45-GPA fusion gene was transformed into *E. coli* MC1061 using the heat shock method. After preparation of the *L. gs*-competent cells, the pMG36e and pMG36e-GPA vectors were transformed into *L. gs* competent cells by electroporation (47). The recombinant *L. gs* is named *Lgs*^36e^ and *Lgs*^GPA^, respectively.

### Expression and purification of the GPA peptide

The recombinant strain *E. coli* BL21 transformed with pMFH-GPA was induced by 0.3 mmol/L β-D-isopropylthiogalactopyranoside (IPTG). The cells were collected by centrifugation at 3,500 × *g* for 20 min. Pelleted cells were resuspended in moderate Tris-HCl buffer (20 mmol/L Tris, 100 mmol/L NaCl, pH 8.0). Then, the cells were lysed by homogenization. The pellets were collected by centrifugation at 12,000 × *g* for 20 min. The pellet was dissolved in 6 mol/L urea (pH 8.0). After sonication, the supernatants were collected by centrifugation at 7,500 × g for 20 min. An equal volume of pre-cooled ethanol (−20°C) was added to precipitate protein. The pellets were collected by centrifugation at 12,000 × *g* for 20 min. The supernatants were detected by tricine sodium dodecyl sulfate-polyacrylamide gel electrophoresis (tricine-SDS-PAGE) (48, 49) or Western blot analysis. After CNBr treatment and cleavage of the fusion protein (50), the GPA peptide was purified by HPLC and detected by Western blot analysis.

### Cell culture and insulin induction analysis

Min6, a mouse islet cell line, was cultured in RPMI Medium 1640 with 10% FBS and cultured at 37°C in 5% CO_2_ in a humidified incubator. After 80%–90% confluency, the cells were seeded in 24-well plates. After 80%–90% confluency, cells were rapidly rinsed twice with Hank’s balanced salt solution (HBSS) (51), incubated by HBSS with 3 mmol/L glucose for 2 h. Then, the cells were treated with the sterile medium of the recombinant *L. gs,* which was diluted with an equal volume of RPMI Medium 1640 for 2 h. Liraglutide (100 nmol/L) mixed with RPMI Medium 1640 was used as a positive control. Then, the supernatants were collected by centrifugation (10 min, 3,000 *× g*, 4°C), and the levels of insulin in the supernatants were detected with insulin enzyme-linked immunosorbent assay (ELISA) kits (Ruixin Co., Quanzhou, China). For the samples of Western blot analysis, the cells were treated with the above mixes for 24 h, then the cells were collected to analyze the expression of PDX-1.

### Animal model experiments

All animals were kept and cared for in accordance with the guidelines of the Hubei University of Technology Animal Care and Use Committee. The animals were fed a standard food diet. When treated with recombinant *L. gs*, mice or rats were gavaged with microbiota solution (1–5 × 10^8^ CFU bacteria) (52).

#### Animal experiment 1

Six Sprague-Dawley (SD) rats (8–10 weeks old) were provided by the Laboratory Animal Center of Huazhong Agricultural University (Wuhan, China). To assess the half-life period of GPA, 0.2 mg/kg of body weight GPA was injected into the tail vein of rat (*n* = 1) for 24 h, and blood samples were prepared via tail vein into a microcentrifuge tube, 500 µL acetonitrile-water (vol/vol: 1:1) was added, and centrifuged for 10 min at 10,000 *× g*. The mix of supernatant was detected by HPLC. To assess the recombinant *L. gs* that can be colonized in the gut and secrete GPA, SD rats were allocated into two groups with matching average body weight: (i) SD rats were oral gavage with *Lgs*^36e^ (*n* = 3) and (ii) SD rats were oral gavage with *Lgs*^GPA^ (*n* = 3). The rats were daily gavaged with *Lgs*^GPA^. After a week, the stool samples of each group were collected, and they were frozen at −80°C for further use. Blood samples for HPLC were prepared on the tenth day and were similar to the above steps.

#### Animal experiment 2

To assess the glucose-lowering activity of GPA, 12 male C57BL/6J mice (10–12 weeks old) were purchased from the Charles River Co. (Beijing, China). The mice were allocated into two groups with matching average body weights: (i) Con group: Mice were intraperitoneally injected with physiological saline for 12 h (*n* = 6), (b) GPA group: Mice were intraperitoneally injected with 0.2 mg/kg of body weight GPA for 24 h. The glucose tolerance test (GTT) was measured after the mice were intragastrically administered 2 g/kg of body weight glucose.

#### Animal experiment 3

To further assess the hypoglycemic effect of GPA, six male *db/db* mice (6–8 weeks old) were purchased from the GemPharmatech Co. (Chengdu, China). Random blood glucose levels (1 h, 3 h, 6 h, 9 h, and 12 h) of *db/db* mice were monitored after the mice were intraperitoneally injected with 0.2 mg/kg of body weight GPA. In addition, we monitored the fasting blood glucose (FBG) levels of the *db/db* mice pre-treatment or post-treatment (24 h) of 0.2 mg/kg of body weight GPA peptide.

#### Animal experiment 4

To further assess the hypoglycemic effect of *Lgs*^GPA^, 11 male *db/db* mice (6–8 weeks old) were obtained from the Cavens Laboratory Animal Technology Co. (Jiangsu, China). The schematic of the animal experiment is shown in Fig. 2a. Mice were allocated into two groups with matching average body weights and blood glucose levels: (i) *db/db* mice were orally gavaged with *Lgs*^36e^ (*n* = 6) and (ii) *db/db* mice were orally gavaged with *Lgs*^GPA^ (*n* = 5). The mice were daily gavaged with *Lgs*, after a week, the stool samples of each group were collected, and they were frozen at −80°C for further use. The FBG levels of *db/db* mice were monitored every week. GTT and insulin tolerance test (ITT) were measured after treatment for 10 weeks.

### GTT and ITT assays

For GTT, mice were fasted for 12 h with free access to autoclaved water, and then they were intragastrically administered glucose at 2 g/kg (C57BL/6J mice) or 1 g/kg (*db/db* mice) of body weight. For ITT, mice were fasted for 6 h with free access to autoclaved water, and then they were intraperitoneally injected with 1.5 U/kg body weight insulin. The blood glucose levels were measured at 0, 15, 30, 60, 90 and 120 min, and the area under the curve (AUC) was calculated after the mice were treated with glucose/insulin (53). A glucometer (Sinocare, China) was used to measure the blood glucose of mice.

### HPLC analysis

HPLC analysis was carried out with the protocol described previously (8) with slight modifications. Briefly, the GPA was separated or detected at a flow rate of 1 mL/min. The gradient program was set as follows: 0–5 min, 95% buffer A (0.1% trifluoracetic acid in acetonitrile), and 5% buffer B (0.1% trifluoracetic acid in H_2_O); 5–30 min, 95%-5% buffer A, 5%-95% buffer B; 30–40 min, 95%–100% buffer A. The peaks were collected, and they were detected by Western blot analysis.

### Biochemical analysis of serum samples

All animals were anesthetized after an overnight fast. Then, blood samples were rapidly collected. After centrifugation (10 min, 3,000 *× g*, 4°C), serum samples were stored at −80°C. The concentrations of alanine aminotransferase (ALT), aspartate transaminase (AST), creatinine (CRE) and urea (BUN), total cholesterol (TC), total triglycerides (TG), high-density lipoprotein cholesterol (HDL-C), low-density lipoprotein cholesterol (LDL-C), and glycosylated hemoglobin A1c (GHbA1c) in serum were measured by corresponding commercial kits (Jiancheng Co., Nanjing, China) according to the instructions, respectively.

### Determination of secreted GPA peptide

The recombinant *Lgs*^36e^ and *Lgs*^GPA^ were amplified in culture, respectively. Clarified supernatants (300 mL) by centrifugation (15 min, 10,000 × *g*, 4°C) were precipitated with 10% trichloroacetic acid for 30 min on ice, and the pellets were washed twice in ice-cold ethanol/ether (1:1), and the pellets were dissolved in 1 × loading buffer (54). The samples were separated by tricine-SDS-PAGE (48, 49). The cell pellets from 3 mL of bacterial culture were collected by centrifugation (15 min, 10,000 × *g*, 4°C). The pellets were added to 300 µL PBS and then lysed by sonication, and they were centrifuged (15 min, 10,000 × *g*, 4°C). The supernatants were added to 1/4 vol 5 × loading buffer and heated at 95°C for 10 min. The samples were separated by tricine-SDS-PAGE. The concentration of GPA in the supernatants secreted by *Lgs*^GPA^ was measured using the GLP-1 ELISA kit (Ruixin Co., Quanzhou, China).

### Western blot analysis

After the above samples were separated by tricine-SDS-PAGE or tricine-SDS-PAGE. For Western blot analysis, electrophoresis gels were blotted onto PVDF membranes (pore size 0.22 µm), and they were blocked with 5% milk. The anti-GLP-1 (1:1,000) or anti-PDX-1 (1:1,000) specific antibodies were used to detect the expression of target proteins or peptides. HRP-conjugated secondary antibodies were utilized for ECL visualization by the fully automatic image analysis system (Biotanon, China).

### 16S rRNA analysis of stool microbiota

Stool samples were collected as described above. They were collected at the same time points (10:00 a.m.) to minimize circadian influences on the microbiome. The compositions of the stool microbiome were sent to Majorbio Biotechnology (Shanghai, China). After DNA extraction, the primer pair 515F and 806R targeting V4 regions (31) of the 16S rRNA gene was used for amplification. The resulting amplicons were purified. The purified products were pooled to construct the Miseq library, and then Miseq sequencing. Subsequently, bioinformatic analysis was processed with the Majorbio Cloud platform. To compare the relative abundance of gut microbiota among different groups, analyses were conducted at the phylum, order, family, and genus levels (55). The raw sequencing data were deposited into the NCBI database (submission ID SUB14383914, BioProject ID PRJNA1102494; submission ID SUB14384293, BioProject ID PRJNA1139654).

### Statistical analysis

All data were presented as mean ± SEM. The unpaired two-tailed Student’s t test was used for two-group comparisons. Pairwise comparisons of microbiota (dis)similarity by LDA and LEfSe analysis were compared using the Mann-Whitney U test. One-way ANOVA followed by Tukey’s multiple comparison test for multiple group comparisons using GraphPad Prism 8 Software (San Diego, CA, USA), and *P < 0.05, **P < 0.01, ***P < 0.001 represented statistically significant.

## Data Availability

Raw reads reported in this paper are deposited in the National Center for Biotechnology Information’s Sequence Read Archive (SRA) database under the BioProject ID numbers PRJNA1102494 and PRJNA1139654.

## SUPPLEMENTAL MATERIAL

Supplemental material spectrum.02828-24-s0001.pdf Tables S1 to S3; Fig. S1 to S10.

ASM does not own the copyrights to Supplemental Material that may be linked to, or accessed through, an article. The authors have granted ASM a non-exclusive, world-wide license to publish the Supplemental Material files. Please contact the corresponding author directly for reuse.
