# Supplementary material for: Oral delivery of GLP-1 peptide using recombinant Lactobacillus gasseri for the treatment of type 2 diabetes mellitus
Source: Microbiol Spectr. 2025 Jun 18;13(8):e02828-24. doi: 10.1128/spectrum.02828-24 (PMC12323307; doi:10.1128/spectrum.02828-24)
Supplement: Supplemental material — Tables S1 to S3; Fig. S1 to S10. [file spectrum.02828-24-s0001.pdf]

Supporting Material for

An engineered probiotic approach for the oral delivery GLP-1 peptide to treat Type-2 diabetes mellitus

Zhiqiang Ke[1,2,3], Qianqian Ma[1,2], Xiaonan Ye[1], Yan Jin[1], Yanlin Wang[1], Xinyuan Zhao[3,*], Zhengding Su[1, 2,4,*]

[1]Protein Engineering and Biopharmaceuticals science, Hubei University of Technology, Wuhan 430068, China.

[2]Institute of Materia Medica, School of Pharmaceutical Sciences, Xinjiang University, Urumqi, 830017, China.

[3]National Demonstration Center for Experimental General Medicine Education, School of Basic Medical Sciences, Hubei University of Science and Technology, Xianning, 437100, China.

[4]Wuhan Biodevelop Inc., Wuhan, 430071, China.

***To whom correspondence may be addressed**: Zhengding Su, Email: james_su@xju.edu.cn or zhengdingsu@hbut.edu.cn, ORCID iD: 0000-0003-3558-001X or Xinyuan Zhao, Email: zhaoxinyuan@hbust.edu.cn, ORCID iD: 0009-0007-1520-8620.

Supplementary method

Construction of pMFH-GPA vector

The GPA gene was amplified from plasmid pUC57-usp45-GPA using primer pair *Eco*R I-GLP-1-F and M13-R. The target gene fragments GPA were digested by *Eco*R I and *Bam*H I restriction enzymes and cloned into the pMFH vector. Then, the ligation mixture was transformed into *E. coli* DH5α to get a stable recombinant plasmid. Positive clones of *E. coli* DH5α transformant were screened by colony PCR using primer pairs *Eco*R I-GLP-1-F and Ptrc-R.

Construction of pMG36e-usp45-GPA vector

Primer pair M13-F and M13-R were used to amplify a 225 bp product 1 from plasmid pUC57-usp45-GPA. The products were digested by *Xba* I and *Pst* I restriction enzymes and cloned into the pMG36e vector. Then, the ligation mixture was transformed into *E. coli* MC1061 to get a stable recombinant plasmid. The plasmid is named pMG36e-GPA-Excess.

In order to eliminate the influence of excess bases between the promoter and the signal peptide on the signal peptide sequence, overlapping PCR technique was used under the following conditions. Pre denaturation at 95 °C, 5 min; denaturation at 95 °C, 1 min; annealing at 55 °C; extension at 72 °C, 45 s; and final extension at 72 °C for 10

min. Primer pair LOOP-F and pMG36e-R were used to amplify a 618 bp product 2 from plasmid pMG36e-GPA-Excess. Primer pair Fusion-F and LOOP-R were used to amplify a 369 bp product 3 from plasmid pMG36e-GPA-Excess. Primer pair Fusion-F and pMG36e-R were used to amplify a mixture of product 1 and 2.

Then, the fusion gene fragments were digested by *Eco*R and *Bam*H I restriction enzymes and cloned into the pMG36e vector. Then, the ligation mixture was transformed into *E. coli* MC1061 to get a stable recombinant plasmid. The plasmid is named pMG36e-GPA.

Preparation of Lactobacillus gasseri (L. gs) competent cells

To prepare the *L. gs* competent cells, the *L. gs* was inoculated into the liquid Man Rogosa Sharpe agar (MRS) medium containing 2.5% glycine, and grown at 37 °C to an OD_{600} nm of 0.3 – 0.4, then the *L. gs* was incubated at 4 °C for 10 min. After incubation, the cells were collected by centrifugation at 5000 × g for 10 min and washed with distilled pre-chilled sterile water by centrifugation at 5000 × g for 10 min. The cells were washed with pre-chilled buffer I (0.5 mol/L sucrose solution containing 10% glycerol) by centrifugation at 8000 × g for 10 min. The cells were washed with pre-cool buffer II (0.5 mol/L sucrose solution, 10% glycerol, and 0.05 mmol/L EDTA) by centrifugation at 8000 × g for 10 min. The cells were incubated at 4 °C for 10 min. Then the cells were washed with pre-chilled buffer I for three times. Finally, the cells were resuspended with pre-chilled buffer I, after sub-packaging, the cells were stored at -

80 °C for further use.

Transformation of plasmids by electroporation

The prepared competent cells and 1 μg plasmid pMG36e or pMG36e-usp45-GPA (pMG36e-GPA) were mixed and transferred to a pre-chilled electroporation cuvette. Then they were exposed to a single electric pulse at 2 KV, 25 μF and 200 Ω using a Gene-Pulser (Bio-Rad, USA). The suspensions were immediately mixed with 1 mL MRS broth containing 2.5% glycine, 5% sucrose, 20 mmol/L $MgCl_2$, and 2 mmol/L $CaCl_2$, and then incubated at 37°C for 3 hr. The incubated cells were spread on the three MRS agar plates containing erythromycin (final concentration of 10 μg/mL) and incubated at 37°C for 36-48 hr. Then positive transformants were selected on MRS agar erythromycin plates, and several of them were confirmed by colony PCR.

Table S1. Stains and plasmids used in this experiment

Strain and plasmid	Relevant feature	Source
Strains		
E. coli DH5α	*E. coli* DH5α (Top 10)	Laboratory stock
E. coli BL21	*E. coli* BL21 (DE3)	Laboratory stock
L. gs	*Lactobacillus gasseri*	Laboratory stock
MC1061	*E. coli* MC1061 (T0003)	MiaoLing Bio, China
MC1061-pMG36e	MC1061 integrated with pMG36e plasmid	This study
MC1061-usp45-GPA	MC1061 integrated with pMG36e-usp45-GPA plasmid	This study
Lgs^{36e}	*L. gs* integrated with pMG36e plasmid	This study
Lgs^{GPA}	*L. gs* integrated with pMG36e- usp45-GPA plasmid	This study
Plasmids		
pMFH-GLP-1	pMFH infused GLP-1(7-37)^{R34} gene	Laboratory stock
pMFH-GPA cluster genes	pMFH infused with GPA cluster genes	This study
pMG36e	pMG36e (P0299)	MiaoLing Bio, China
pUC57-usp45-GPA	pUC57 infused with usp45-GPA cluster genes	GenScript Co., China
pMG36e-usp45-GPA	pMG36e-usp45-GPA infused with usp45-GPA cluster genes	This study

The usp45-GPA cluster genes which contains GLP-1 gene, PTD gene, ABP gene, usp45 signal peptide sequences gene and linkers were synthesized after codon optimization. GPA: GLP-1-PTD-ABP. PTD: protein transduction domain. ABP: serum albumin binding peptide.

Table S2. Primers used in this study

Primers for PCR amplification and sequencing	sequence (5'-3')
M13-F	TGTAAAACGACGGCCAGT
M13-R	CAGGAAACAGCTATGACC
pMG36e-F	AGGTAGGTAAAAAAATATTCG
pMG36e-R	GTACCGTCGCCTTTACCAACT
*Eco*R I-GLP-1-F	CCGGAATTCATGCATGCCGAAGGAACCTTT
LOOP-F	CGGAGGAATTTTGAAATGAAAAAAAAGTTGATATCTAGT
LOOP-R	ACTAGATATCAACTTTTTTTTCATTTCAAAATTCCTCCG
Ptrc-R	ATAAGAATGCGGCCGCGGGTTATTGTCTCATGAGCGG
Fusion-F	CAAGGGTAAAATGGCCTTTTCCTG
515F	GTGCCAGCMGCCGCGG
806R	GGACTACHVGGGTWTCTAAT

Table S3. Physical and chemical parameters for related protein or peptide

protein or peptide	Number of amino acids	Molecular weight	Theoretical pI
MFH	120	13407.5	9.48
GPA	70	8088.02	10.93
pMFH-GPA	190	21477.58	9.83

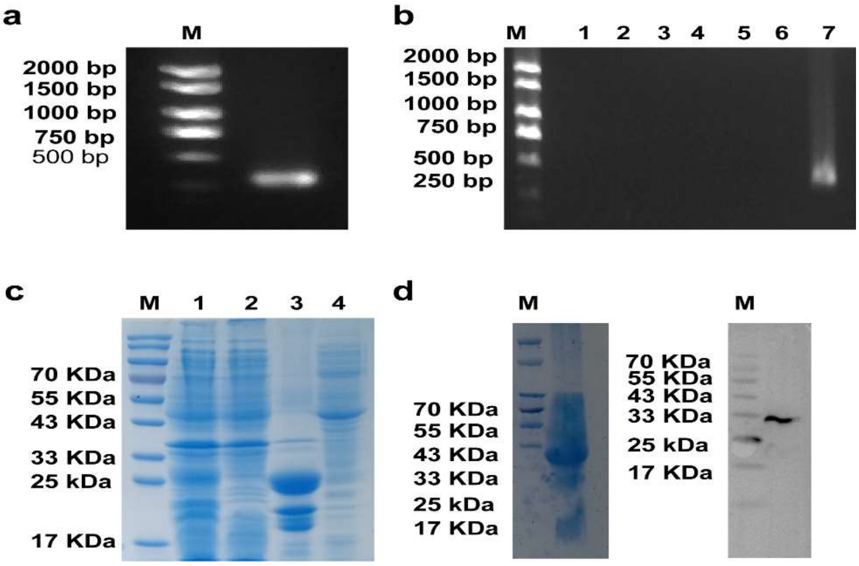

Figure S1. Preparation and characterization of recombinant GPA. (a) Primer pairs *Eco*R1-GLP-1-F and M13-R were used to amplify a product (266 bp) from plasmid pUC57-usp45-GPA. (b) Positive clones of *E. coli* DH5α transformant were screened by colony PCR using primer pairs *Eco*R1-GLP-1-F and Ptrc-R. The lane 7 is a positive clone. (c) SDS–PAGE analysis of the expressed fusion proteins containing GPA. Lane M: molecular weight markers. Lane 1: cell lysate with Tris-HCl buffer (20 mM Tris and 100 mM NaCl). The total protein of cells harboring pMFH-GPA was prepared by dissolving the cell pellets in the Tris-HCl buffer. Lane 2: The supernatant after 12000 g centrifugation of cell lysate. Lane 3: The supernatant after 7500 g centrifugation of urea (6 mol/L) dissolved inclusion bodies. Lane 4: The Ni-NTA elution sample with buffer B (6 mol/L urea and 500 mmol/L imidazole). (d) The SDS–PAGE (left) and Western blot (right) analysis of the protein of pMFH-GPA was prepared by SDS sample buffer. GPA: GLP-1-PTD-ABP. PTD: protein transduction domain. ABP: serum albumin binding peptide.

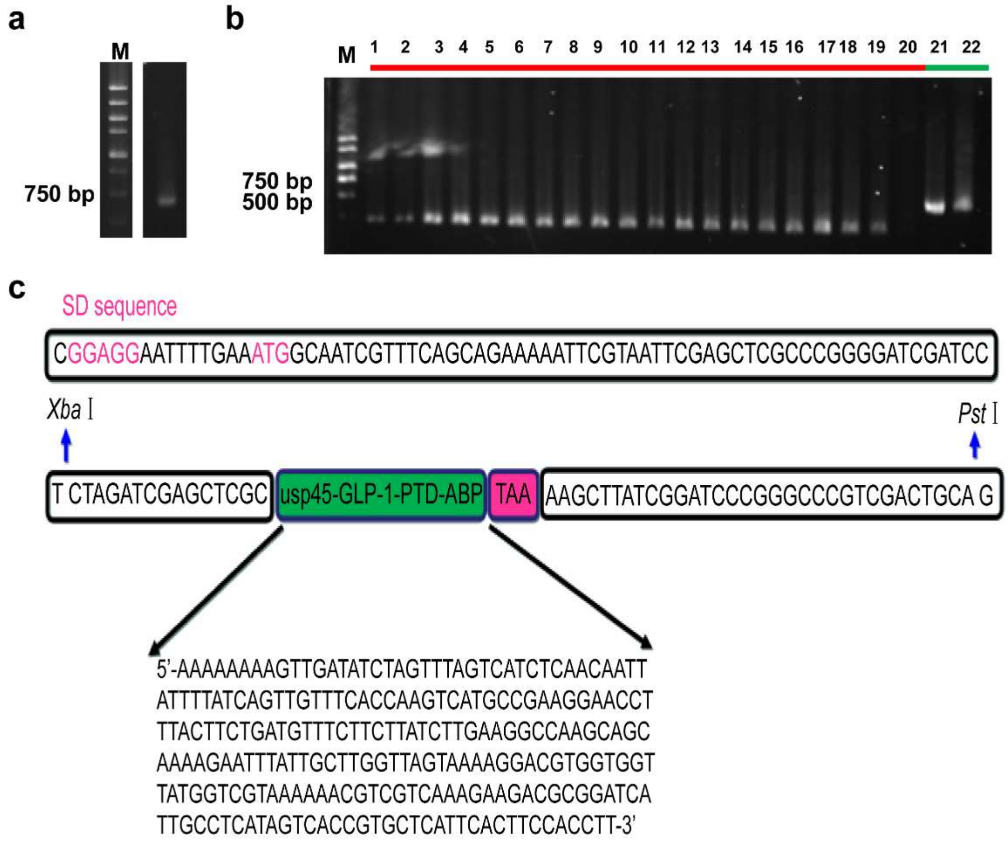

Figure S2. Construction of pMG36e-usp45-GPA-Excess plasmids. (a) Primer pairs M13-F and M13-R were used to amplify a product 1 (225 bp) from plasmid pUC57-usp45-GPA. (b) Positive clones of *E. coli* MC1061 transformant were screened by colony PCR using primer pairs pMG36e-F and pMG36e-R. The lanes 21 and 22 are two positive clones. (c) Sequence analysis of pMG36e-usp45-GPA-Excess plasmids. Lane M: DNA Ladders. GPA: GLP-1-PTD-ABP. PTD: protein transduction domain. ABP: serum albumin binding peptide.

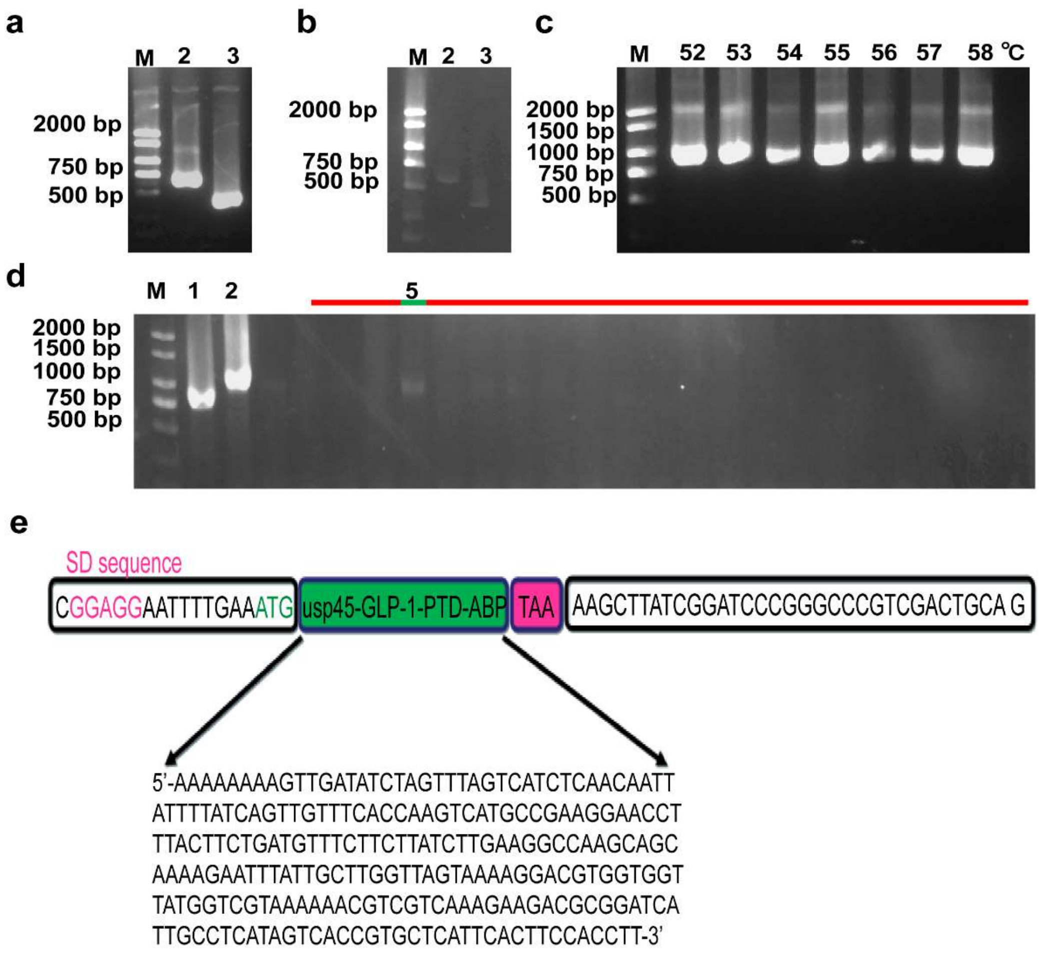

Figure S3. The overlapping PCR technique was used to eliminate the influence of excess bases between the promoter and the signal peptide. (a) Primer pairs LOOP-F and pMG36e-R were used to amplify a 618 bp fragment from plasmid pMG36e-GPA-Excess (product 2). Primer pairs Fusion-F and LOOP-R were used to amplify a 369 bp fragment from plasmid pMG36e-GPA-Excess (product 3). (b) Electrophoretic analysis of products 2 and 3 after DNA gel extraction. (c) Primer pairs Fusion-F and pMG36e-R were used to amplify a 947 bp fragment from product 1 and 2 with gradient PCR. (d) Positive clones of *E. coli* MC1061 transformant were screened by colony PCR using primer pairs Fusion-F and pMG36e-R. The template of lane 1 is the vector pMG36e,

the template of lane 2 is the vector pMG36e-GPA-Excess. The lane 5 is a positive clone.

(e) Sequence analysis of the vector pMG36e-usp45-GPA (pMG36e-GPA). GPA: GLP-1-PTD-ABP. PTD: protein transduction domain. ABP: serum albumin binding peptide.

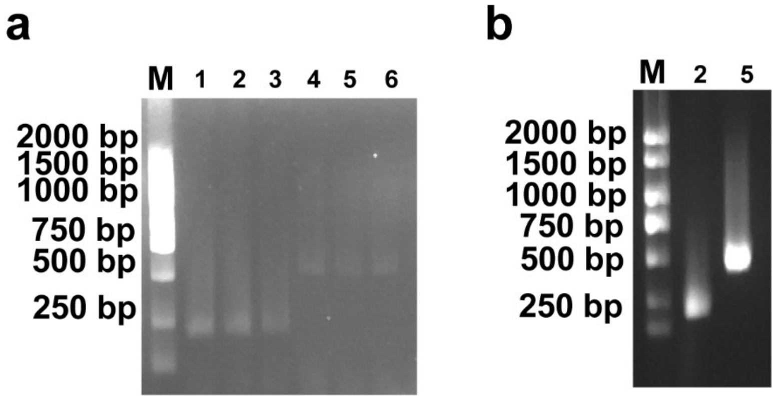

Figure S4. Positive transformants of expressing pMG36e-GPA in *L. gs*. (a) The positive clones of *L. gs* transformant were screened by colony PCR using primer pairs pMG36e-F and pMG36e-R. (b) Primer pairs pMG36e-F and pMG36e-R were used to amplify fragments. The templates of lane 2 and 5 are the plasmids from 2# and 5# colones, respectively. Lane M: molecular weight markers or DNA ladders.

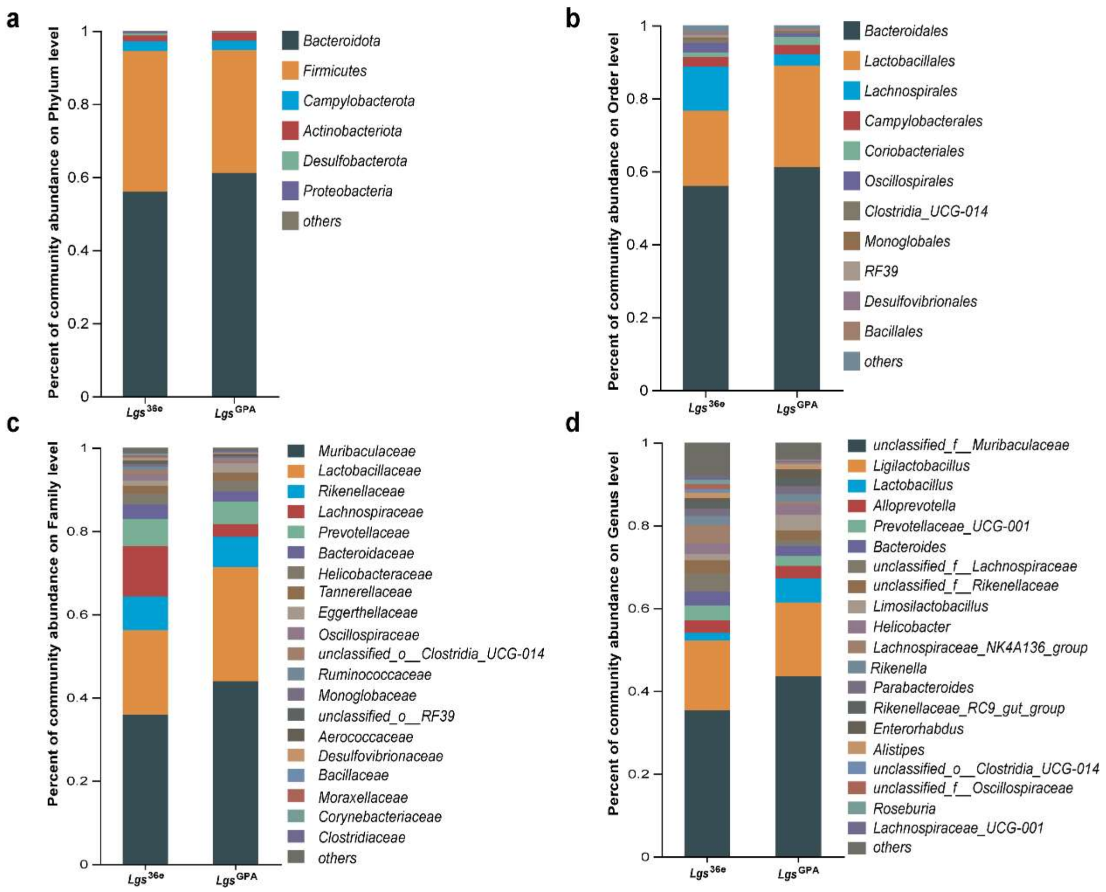

Figure S5. The effects of *Lgs*^{GPA} on the abundance on stool microbiome in *db/db* mice. (a) Community bar plot of the most relevant taxa responsible for the difference at the phylum levels. (b) Community bar plot of the most relevant taxa responsible for the difference at the order levels. (c) Community bar plot of the most relevant taxa responsible for the difference at the family levels. (d) Community bar plot of the most relevant taxa responsible for the difference at the genus levels.

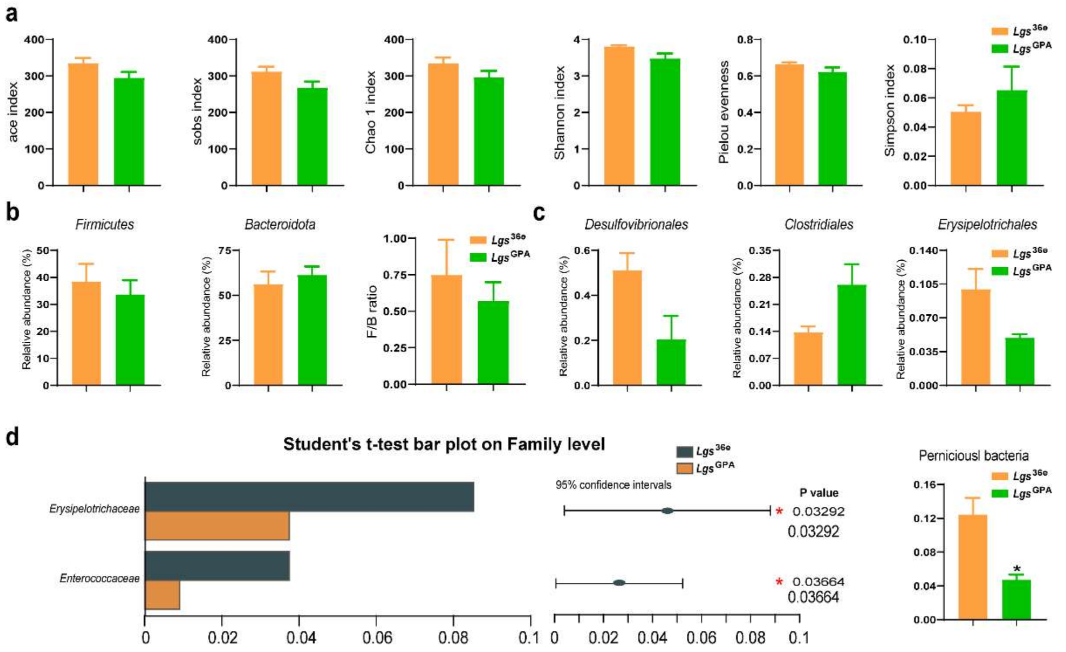

Figure S6. Effects of $\textit{Lgs}^{\mathrm{GPA}}$ on the abundance of stool microbiome in *db/db* mice. (a) α diversity analysis of the ace index, sobs index, Chao1 index, Shannon index, Pielou evenness and Simpson index at the OTU level. (b) At the phylum levels, the relative abundance of *Firmicutes* and *Bacteroidota*, and the ratio of *Firmicutes*/*Bacteroidota* (F/B). (c) At the order levels. The relative abundance of *Desulfovibrionales* (p=0.08), *Clostridiales* (p=0.09) and *Erysipelotrichales* (p=0.08). (d) Community bar plot at the family levels. The relative abundance of *Erysipelotrichaceae*, *Enterococcaceae*, or a sum relative abundance of these two perniciousl bacteria. Data are presented as means ± SEM. Student's t test (a–e, respectively); *$p < 0.05$ vs $\textit{Lgs}^{\mathrm{36e}}$ group.

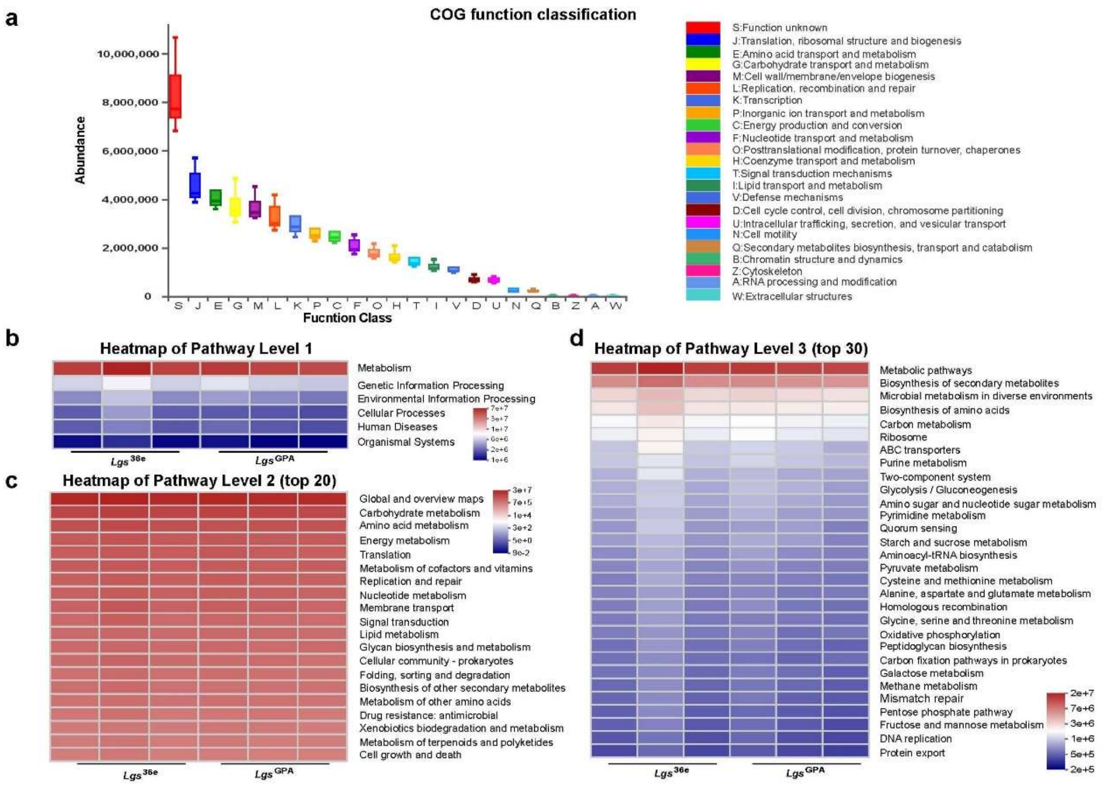

Figure S7. Functional predictive analysis of bacterial microbiota at *db/db* mice. (a) PICRUSt (Phylogenetic Investigation of Communities by Reconstruction of Unobserved States) 2 combined with the Databases of Clusters of Orthologous Genes (COGs) database to predict the function of gut microbiome in *db/db* mice. (b-d) Heatmap of the KEGG Pathway Level 1 (b), 2 (top 20) (c) and 3 (d) (top 20) by PICRUSt2 combined with the KEGG database to predict the function of gut microbiome in *db/db* mice.

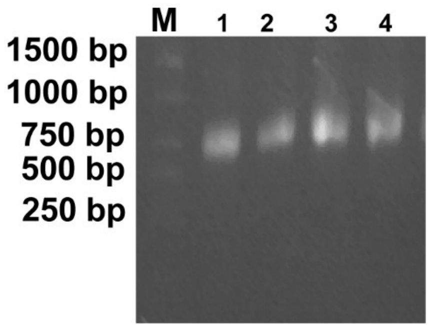

Figure S8. Confirm the stability of the recombinant *L. gs* which were cultured three generation in the stool by PCR using primer pairs Fusion-F and pMG36e-R. The template of lane 1 and 2 are the plasmid from the stool of rats which were gavaged *Lgs*^{36e}. The template of lane 1 and 2 are the plasmid from the stool of rats which were gavaged *Lgs*^{GPA}.

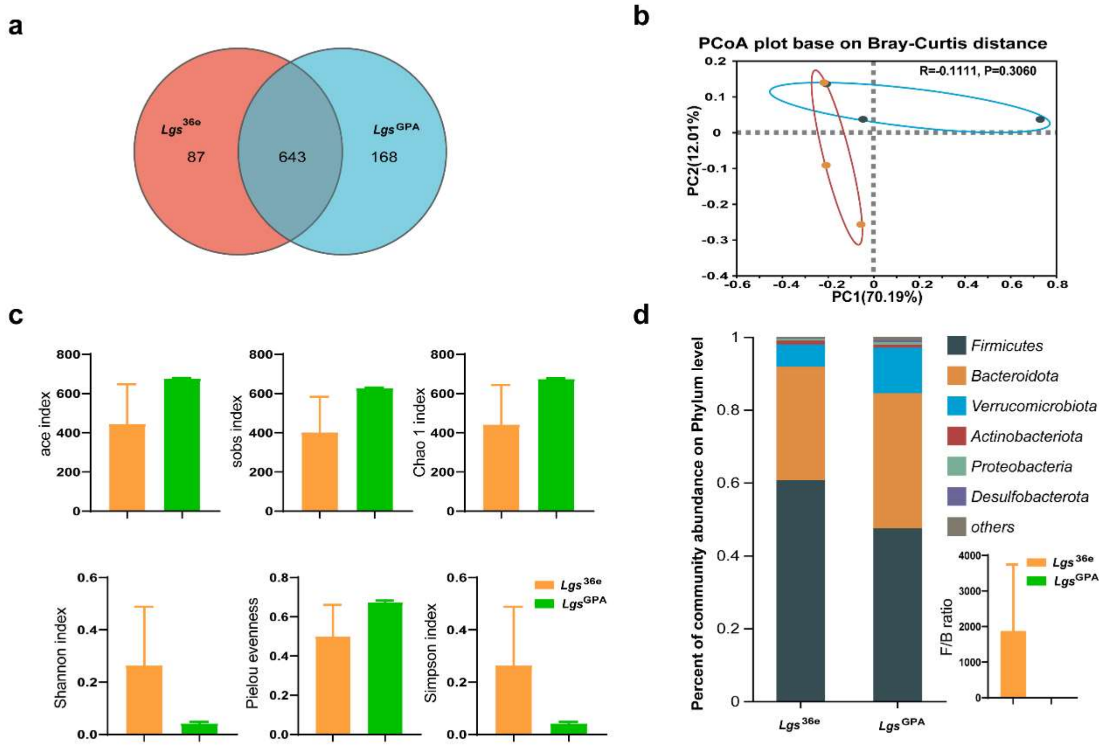

Figure S9. The effects of *Lgs*^{GPA} on the abundance of stool microbiome in SD rats. (a) The total number of core operational taxonomic units (OTUs) shared and that are unique in the Venn diagram. (b) β diversity analysis of principal coordinate analysis (PCoA) analysis on OTU level. (c) α diversity analysis of the ace index, sobs index, Chao1 index, Shannon index, Pielou evenness and Simpson index at the OTU level. (d) At the phylum levels, community bar plot of the most relevant taxa responsible for the difference, and the ratio of *Firmicutes*/*Bacteroidota* (F/B). Data are presented as means ± SEM. Student's t test (c–f, respectively); *$p < 0.05$ vs *Lgs*^{36e} group.

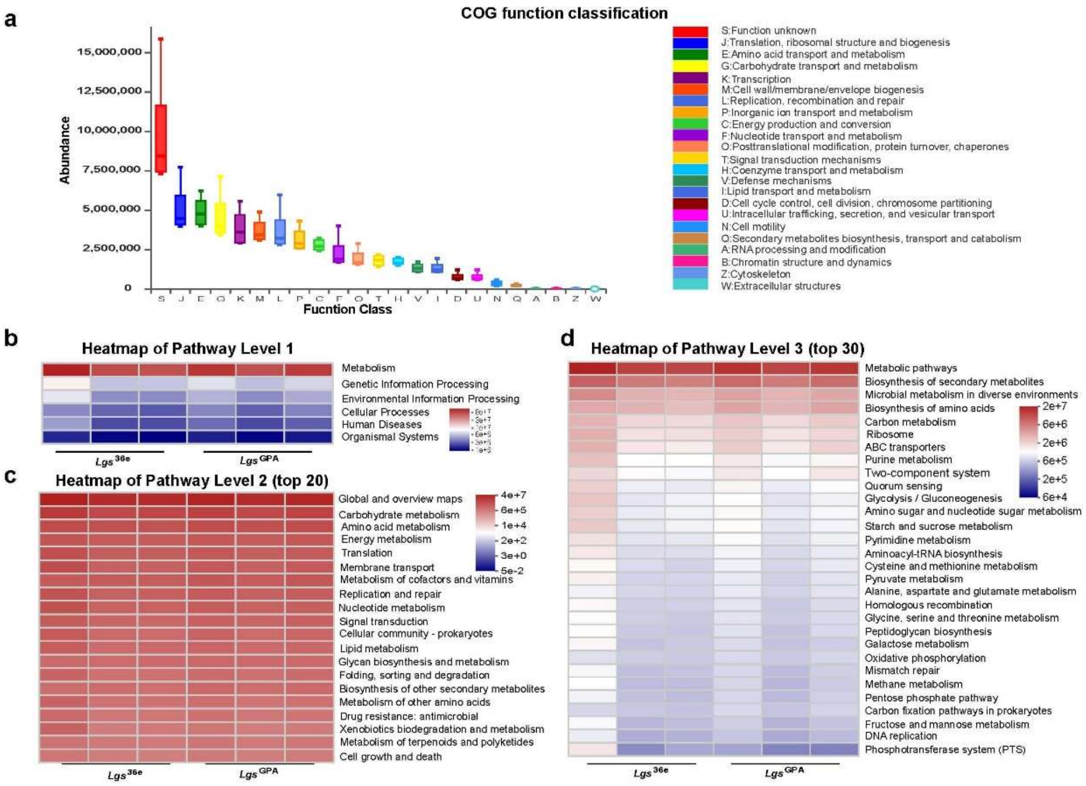

Figure S10. Functional predictive analysis of bacterial microbiota in SD rats. (a) PICRUSt (Phylogenetic Investigation of Communities by Reconstruction of Unobserved States) 2 combined with the Databases of Clusters of Orthologous Genes (COGs) database to predict the function of gut microbiome in SD rats. (b-d) Heatmap of the KEGG Pathway Level 1 (b), 2 (top 20) (c) and 3 (d) (top 20) by PICRUSt2 combined with the KEGG database to predict the function of gut microbiome in SD rats.
